# Comprehensive Analysis of the Transcriptome-Wide m^6^A Methylome in Shaziling Pig Testicular Development

**DOI:** 10.3390/ijms241914475

**Published:** 2023-09-23

**Authors:** Chujie Chen, Xiangwei Tang, Saina Yan, Anqi Yang, Jiaojiao Xiang, Yanhong Deng, Yulong Yin, Bin Chen, Jingjing Gu

**Affiliations:** 1 College of Animal Science and Technology, Hunan Agricultural University, Changsha 410128, China; chencj1995@163.com (C.C.); txw@stu.hunau.edu.cn (X.T.); yansaina99@126.com (S.Y.); yanganqi90@126.com (A.Y.); xjj747955@163.com (J.X.); 17872327665@163.com (Y.D.); yinyulong@isa.ac.cn (Y.Y.); 2 Hunan Provincial Key Laboratory for Genetic Improvement of Domestic Animal, Hunan Agricultural University, Changsha 410128, China; 3 Key Laboratory of Agro-Ecological Processes in Subtropical Region, Institute of Subtropical Agriculture, Chinese Academy of Sciences, Changsha 410125, China

**Keywords:** mRNA m^6^A methylation, testis, *ALKBH5*, *SOX9*, Shaziling pig

## Abstract

RNA *N*^6^-methyladenosine (m^6^A) modification is one of the principal post-transcriptional modifications and plays a dynamic role in testicular development and spermatogenesis. However, the role of m^6^A in porcine testis is understudied. Here, we performed a comprehensive analysis of the m^6^A transcriptome-wide profile in Shaziling pig testes at birth, puberty, and maturity. We analyzed the total transcriptome m^6^A profile and found that the m^6^A patterns were highly distinct in terms of the modification of the transcriptomes during porcine testis development. We found that key m^6^A methylated genes (*AURKC*, *OVOL*, *SOX8*, *ACVR2A*, and *SPATA46*) were highly enriched during spermatogenesis and identified in spermatogenesis-related KEGG pathways, including Wnt, cAMP, mTOR, AMPK, PI3K-Akt, and spliceosome. Our findings indicated that m^6^A methylations are involved in the complex yet well-organized post-transcriptional regulation of porcine testicular development and spermatogenesis. We found that the m^6^A eraser *ALKBH5* negatively regulated the proliferation of immature porcine Sertoli cells. Furthermore, we proposed a novel mechanism of m^6^A modification during testicular development: *ALKBH5* regulated the RNA methylation level and gene expression of *SOX9* mRNA. In addition to serving as a potential target for improving boar reproduction, our findings contributed to the further understanding of the regulation of m^6^A modifications in male reproduction.

## 1. Introduction

RNA *N*^6^-methyladenosine (m^6^A) is a dynamic and reversible mRNA modification that has received a great deal of attention in recent years as a result of its involvement in numerous biological processes [1]. Demethylases (also referred to as “erasers”) and methyltransferases (“writers”) co-regulate m^6^A levels, and m^6^A reader proteins (“readers”) recognize m^6^A-modified mRNAs. These three forms of methylation-modifying effector proteins cooperatively function to perform essential functions in mRNA metabolism, including mRNA alternative splicing, mRNA exporting, mRNA stability, and mRNA translation efficiency, in addition to accelerating mRNA decay [2].

As a fundamental male reproductive organ, the testis is essential for spermatogenesis. Spermatogenesis is a continuous and dynamic process in males that involves the complex transformation of diploid spermatogonial stem cells (SSCs) into haploid spermatozoa [3,4]. Sertoli cells, the only somatic cells present in seminiferous tubules, are a vital component of the SSC niche during spermatogenesis, as they provide vital growth factors and chemokines to developing germ cells [5]. Cumulative comprehensive studies elucidated that m^6^A plays a dynamic role in the regulation of post-transcriptional gene expression and is closely associated with testicular development and spermatogenesis in both humans and mice [6,7,8,9,10]. The investigation of m^6^A modifications in testicular tissues during testicular development in domesticated animals was only recently initiated by researchers. To date, to our best knowledge, the genome-wide m^6^A methylation modification profiles were only obtained from the whole testicular tissues of cattle [11], yak [12], and cattle–yak hybrids [13].

Domestic pigs are economically important meat-producing animals and have many advantages as animal models for human diseases due to their anatomical similarity to humans [14]. Shaziling pigs, a local pig breed that is native to Central China, reach male puberty at approximately 75 days and sexual maturity at around 150 days [15]. Due to their early sexual maturity, Shaziling boars can be an ideal biomedical model for investigating testicular development. However, the role of m^6^A modification in Shaziling pig testes is understudied [16].

In this study, samples of testicular tissue from Shaziling boars were collected at three crucial stages of testicular development: birth, puberty, and maturity. Using methylated RNA immunoprecipitation sequencing (MeRIP-Seq) and RNA sequencing (RNA-Seq), we aimed to resolve the patterns of m^6^A modification and gene expression alterations during testicular development in Shaziling pigs by conducting a thorough analysis of the sequencing data. Based on the results of our analysis, we then selected the immature porcine Sertoli cells as a cell model to simulate the development of somatic cells in the porcine testis and attempted to investigate the effects of silencing *ALKBH5* (a testis-specific demethylase) in immature porcine Sertoli cells on cell proliferation. Furthermore, we concentrated on revealing whether the mRNA expression level and m^6^A deposition level of *SOX9* (a key function marker gene of Sertoli cells) were regulated in an *ALKBH5*-dependent manner. We proposed a novel mechanism of m^6^A modification during testicular development: *ALKBH5* regulated the RNA methylation level and gene expression of *SOX9* mRNA and negatively regulated the proliferation of immature porcine Sertoli cells in vitro. Our study explored the testicular development in Shaziling pigs at the level of RNA m^6^A modifications, provided valuable data for studying testicular development, and deepened our understanding of the role of epigenetic modifications during male sexual maturation.

## 2. Results

### 2.1. Global m^6^A Content, Sequencing Quality Control, and Reference Genome Comparisons at Different Stages of Porcine Testis Development

**Figure 1:**
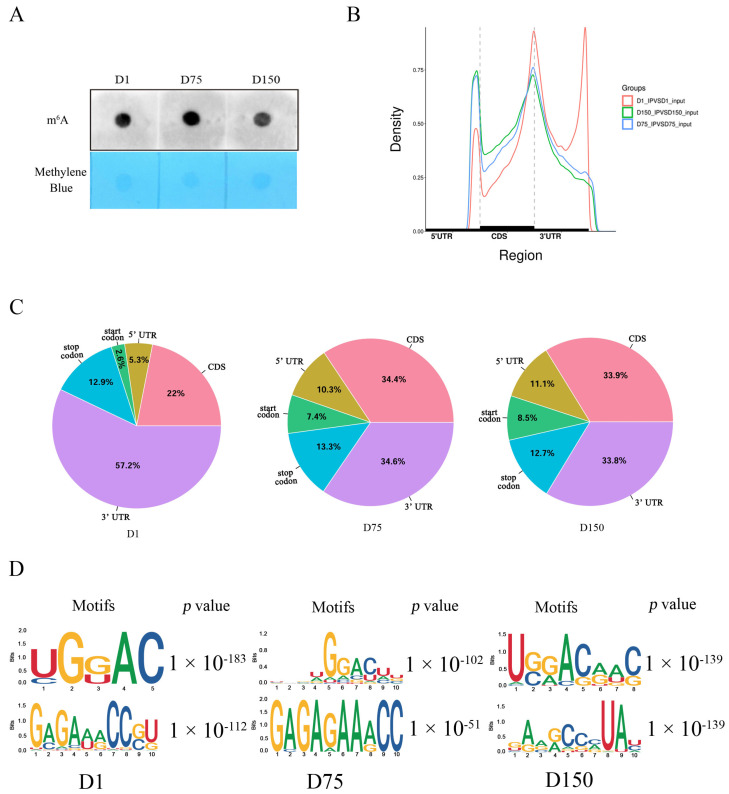
Global m^6^A levels and overview of m^6^A methylation profiles in porcine testes. (**A**) m^6^A dot blot detection of the global m^6^A modification levels in 1-day-old (D1), 75-days-old (D75), and 150-days-old (D150) Shaziling boar testes (*n* = 3). (**B**) Metagene plots demonstrating the regions of m^6^A peaks identified throughout the transcripts genome-wide in the D1, D75, and D150 groups. (**C**) Pie charts illustrating the distribution of m^6^A peaks in mRNA gene structures. (**D**) Top motifs enriched with m^6^A peaks in the D1, D75, and D150 groups.

Throughout the process of rearing Shaziling pigs, we found that at 75 days of age, the boars typically reach puberty, and at 150 days, they are fully sexually mature and can be used to mate. We compared the levels of global m^6^A methylation in three testicular development stages, which were 1 day old (D1, neonatal), 75 days old (D75, pubertal), and 150 days old (D150, mature), using three biological replicates for each developmental stage. An RNA m^6^A dot blot assay revealed that the global m^6^A methylation level of the testicular tissues increased from birth (D1) to puberty (D75) and then decreased at sexual maturity (D150) (Figure 1A).

For MeRIP-Seq and RNA-Seq experiments, three biological replicates of each group of pig testes (D1 neonatal, D75 pubertal, and D150 mature) were utilized. After quality control, we obtained clean data ranging from 5.49 to 7.25 Gb per sample (a total of 117.47 Gb of clean data). In this clean data, the average Q20 and Q30 base distributions were 97.88% and 94.03%, respectively. Clean sequence reads were then aligned to the porcine reference genome (Sscrofa11.1 ftp://ftp.ensembl.org/pub/release-104/fasta/sus_scrofa/dna/ accessed on 15 March 2023), with alignment rates ranging from 82.55% to 90.96% (Supplementary Table S1).

### 2.2. Overview of the m^6^A Methylation Map

Using high-throughput sequencing, we obtained the whole transcriptome m^6^A profiles of boar testicular tissues at three critical developmental stages and identified the genome-wide m^6^A methylation peaks (Supplementary Table S2). According to the genome-wide patterns, the distribution of m^6^A peaks across chromosomes in the pig genome was not uniform. Pig chromosome 1 contained the majority of m^6^A peaks in all three groups (Supplementary Figure S1). We then separated the transcripts into 3′ untranslated regions (UTR), CDS, 5′ UTR, start codon, and stop codon to determine the regions where peaks were located. With increasing days of age, the preferential region of the m^6^A peak distribution shifted from 3′ UTR to CDS (Figure 1B,C). Based on the results of motif analysis, we found that the identified significant m^6^A peaks showed the classic m^6^A RRACH (R = A or G; H = A, C, or U) consensus sequences (Figure 1D).

### 2.3. Genes Enriched with m^6^A Modifications Participated in Significant Biological Processes

**Figure 2:**
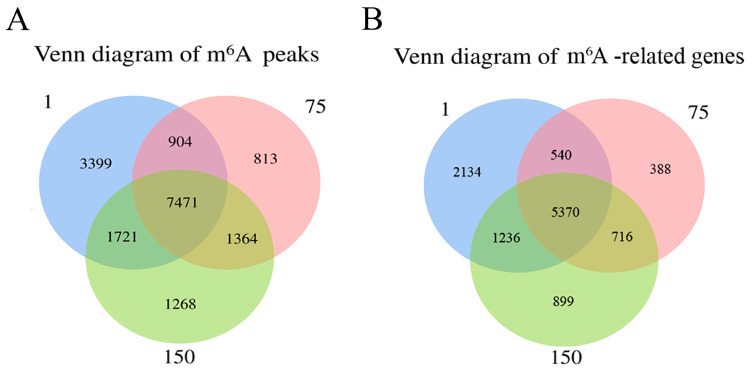

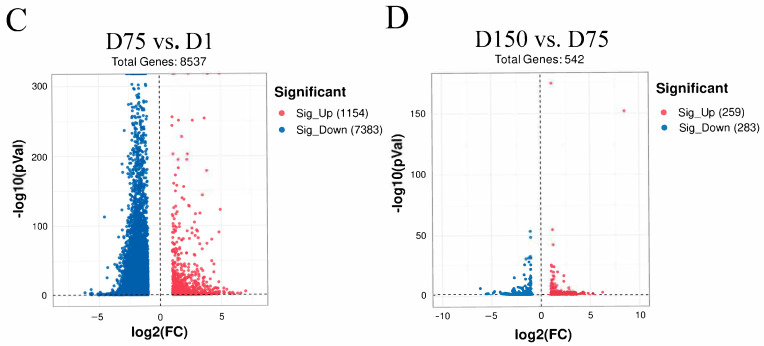
Transcriptome-wide m^6^A analysis in porcine testes. (**A**) The number of shared and unique m^6^A peaks in three groups. (**B**) The Venn diagram shows the number of m^6^A-related genes in three groups. (**C**) Volcano plots showing the significantly differential m^6^A peaks compared between the D75 and D1 groups and (**D**) between the D150 and D75 groups.

In total, 13,495, 10,552, and 11,824 methylated peaks were detected in the D1, D75, and D150 groups, respectively, reflecting the differences in m^6^A modification trends during the development of the porcine testis. Moreover, 7471 peaks were consistently observed in three groups (Figure 2A), of which 5370 genes were modified by m^6^A (Figure 2B). Then, we analyzed the genes containing differential m^6^A peaks for the adjacent developmental stages to gain further insight into the role of m^6^A in porcine testicular development. We discovered that 1154 differentially methylated genes (DMGs) between D1 and D75 were up-methylated, whereas 7383 were down-methylated (Figure 2C). Compared to D75, D150 contained 259 up-methylated and 283 down-methylated genes (Figure 2D). GO and KEGG analyses revealed that up-methylated genes in D75 (compared to D1) were involved in spermatogenesis and the MAPK signaling pathway, while down-methylated genes were predominantly involved in the positive regulation of transcription by RNA polymerase II, signal transduction, spliceosomes, and the metabolic pathways (Supplementary Figure S2). Moreover, the up-methylated genes in D150 (compared to D75) were involved in the positive regulation of the ERK1 and ERK2 cascades and the PI3K-Akt, HIF-1, and TGF-beta signaling pathways. The majority of the down-methylated genes in D150 were involved in the proliferation of G protein-coupled receptor signaling and the inflammatory response (Supplementary Figure S3). The findings indicated that m^6^A modifications were dynamic in porcine testicular tissue, suggesting a crucial role in testicular development.

### 2.4. Gene Expressions during Porcine Testicular Development

To further investigate the regulatory roles of m^6^A on gene expression, we performed an RNA-Seq analysis on testicular tissues at three developmental stages. We examined differentially expressed genes (DEGs) between adjacent developmental stages. Between D1 and D75, a total of 9201 DEGs were identified, of which 4467 were up-regulated and 4734 were down-regulated. Comparing D150 to D75, there were 910 up-regulated genes and 608 down-regulated genes (Supplementary Figure S4).

Enrichment analysis revealed that for D75, the up-regulated genes (compared to D1) mainly participated in protein binding, spermatogenesis, and spermatid development, whereas the down-regulated genes were primarily involved in protein kinase binding, oxidoreductase activity, and intracellular signal transduction (Supplementary Figure S5). For D150 (compared to D75), the up-regulated genes were mainly involved in intracellular signal transduction, spermatogenesis, and butanoate metabolism, while the down-regulated genes regulated the endoplasmic reticulum, the collagen-containing extracellular matrix, and ECM–receptor interaction (Supplementary Figure S6). Given the gene expression patterns during porcine testicular development, we proposed that DEGs may play important roles in the testicular development of pigs.

### 2.5. Conjoint MeRIP-Seq and RNA-Seq Analysis

To investigate the synergistic patterns of m^6^A modifications and the regulation of gene expression in porcine testes, we performed a conjoint analysis of the MeRIP-Seq and RNA-Seq data. We found that the m^6^A peaks showed a significantly positive correlation with gene expression during testicular development from birth to puberty (D75 vs. D1, Spearman correlation *p* < 0.001). Although a negative correlation between the differential methylation peaks and gene expression levels from puberty to maturity (D150 vs. D75) was observed, it was not significant.

**Figure 3:**
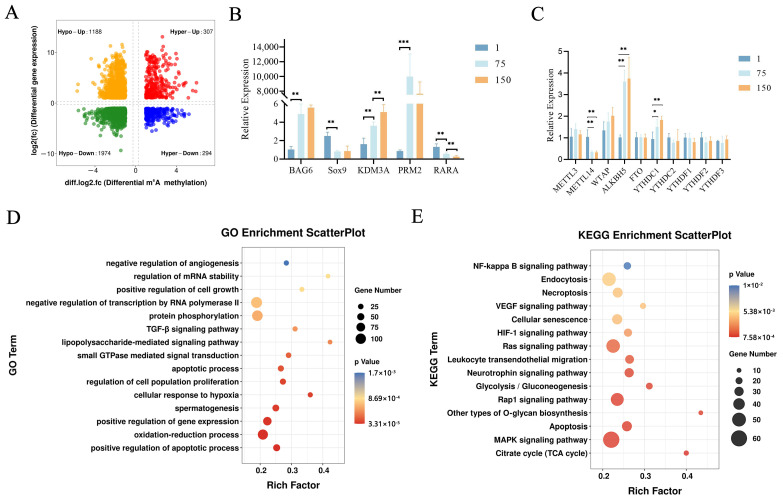
Conjoint analysis of m^6^A-Seq and RNA-Seq data in porcine testes. (**A**) Four quadrant plots showing differentially expressed genes with differentially methylated m^6^A peaks (|log2.fc| ≥ 1, *p* < 0.05) among the studied groups. (**B**) The RT-qPCR analysis of five genes (*BAG6*, *SOX9*, *KDM3A*, *PRM2*, and *RARA*) and (**C**) core m^6^A methylation-related genes in three groups (D1, D75, and D150) determined the relative mRNA levels. *, ** and *** represent *p* < 0.05, *p* < 0.01, and *p* < 0.001 respectively. (**D**) GO enrichment analysis of Diff_1 gene set in D75 vs. D1. (**E**) KEGG pathway enrichment analysis of Diff_1 gene set in D75 vs. D1.

Subsequently, we focused on the developmental stage from birth to puberty. From D1 to D75, 3763 differentially expressed and synchronously differentially methylated genes were identified by a conjoint analysis of the MeRIP-Seq and RNA-Seq data, which are referred to here as Diff_1 (Supplementary Table S3). The Diff_1 genes were subsequently divided into four sections, including 307 hypermethylated and up-regulated genes (hyper-up), 294 hypermethylated and down-regulated genes (hyper-down), 1188 hypomethylated and up-regulated genes (hypo-up), and 1974 hypomethylated and down-regulated genes (hypo-down) (Figure 3A).

To confirm the accuracy of our analysis, we randomly selected 5 genes (*BAG6*, *SOX9*, *KDM3A*, *PRM2,* and *RARA*) involved in spermatogenesis, and 10 core m^6^A methylation genes, including methyltransferases (*METTL3*, *METTL14*, and *WTAP*), demethylases (*FTO* and *ALKBH5*), and m^6^A readers (*YTHDC1*, *YTHDC2*, *YTHDF1*, *YTHDF2*, and *YTHDF3*) for RT-qPCRs to validate our sequencing data. The expression levels of *SOX9* and *RARA* decreased with age, while the expression levels of *KDM3A* increased with age, and the expression levels of *PRM2* first showed a trend of increasing and then decreasing (Figure 3B). The changes in the gene expression levels of the core methylation genes showed different trends in three testicular development stages. However, we only observed significant up-regulations in *ALKBH5* and *YTHDC1*, whereas *METTL14* demonstrated a significant down-regulation (Figure 3C). The above results were consistent with our transcriptome analysis and reflected the validity of our results.

To further uncover the biological significance of the dynamically m^6^A-modified genes, we performed a functional enrichment analysis of the Diff_1 gene set (D75 vs. D1). The top significant GO terms are shown in Figure 3D. The Diff_1 genes were mainly enriched for the regulation of cell apoptosis and proliferation, spermatogenesis, the positive regulation of gene expression, the oxidation reduction process, and the response to hypoxia. The results of the KEGG analysis showed that the Diff_1 genes were mainly involved in MAPK, Rap1, the RAS signaling pathway, apoptosis, the citrate cycle, and glycolysis (Figure 3E). In D150 vs. D75, we found 96 differentially expressed and simultaneously differentially methylated genes (referred to as Diff_2). The Diff_2 genes mainly participated in transmembrane transport and the Wnt signaling pathway (Supplementary Figures S7 and S8; Supplementary Table S4). Collectively, these results suggested that m^6^A may regulate gene expression and be involved in spermatogenesis in porcine testes.

### 2.6. Effects of ALKBH5 Silencing in Immature Porcine Sertoli Cells (iSCs)

**Figure 4:**
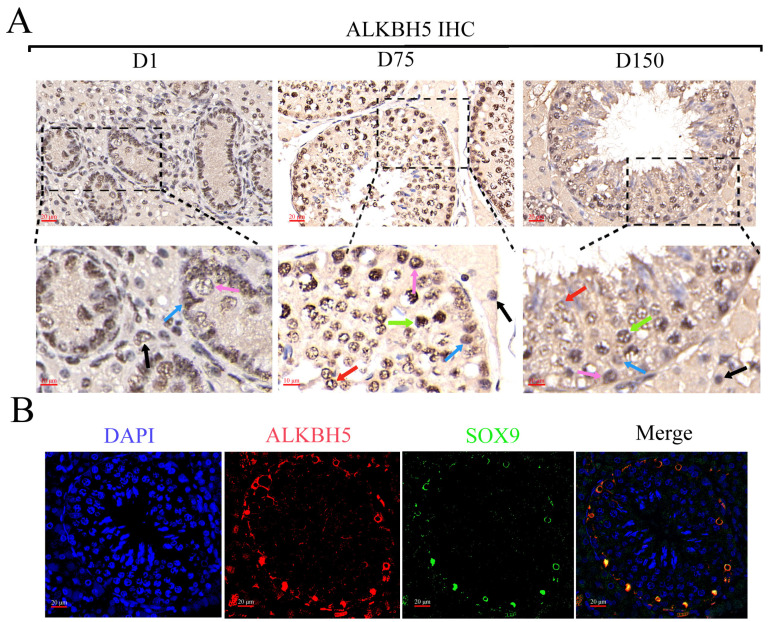

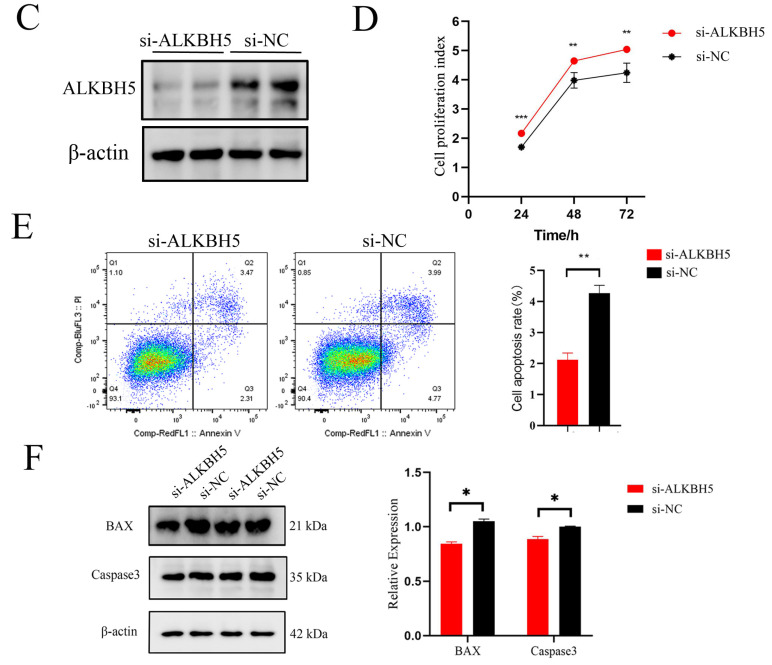
The biological profile and potential mechanisms of *ALKBH5* in iSCs. (**A**) The immunohistochemical (IHC) analysis of ALKBH5 expressions in the testes of Shaziling pigs at different ages (D1, D75, and D150) and cell types are pointed out by arrows of various colors (*n* = 3). Black arrow, Leydig cells; blue arrow, Sertoli cells; pink arrow, spermatogonia; green arrow, spermatocytes; red arrow, spermatids; (**B**) Immunofluorescence (IF) analysis showed colocalization of ALKBH5 expression with SOX9 (a marker of Sertoli cells) in Shaziling pig testes at D150 (*n* = 3). (**C**) Western blot analysis detected the expression of ALKBH5 after silencing. β-actin was used as an internal control. (**D**) CCK-8 assay estimated the effects of *ALKBH5* silencing on cell viability in iSCs. (**E**) Annexin-V/PI double staining assay evaluated the cell apoptosis rates after *ALKBH5* silencing. (**F**) Western blot analysis detected the expression of BAX and Caspase-3 after *ALKBH5* silencing. β-actin was used as an internal control. *, ** and *** represent *p* < 0.05, *p* < 0.01, and *p* < 0.001 respectively.

Once the distinct profiles of m^6^A in the three developmental stages of porcine testes were analyzed, we further clarified the biological profile and potential mechanisms of *ALKBH5* during testicular development using iSCs in vitro. To understand the expression patterns and distribution of ALKBH5 in porcine testes, we first detected the expression of ALKBH5 in neonatal, pubertal, and mature Shaziling pig testes. IHC staining confirmed a high ALKBH5 level in almost every kind of cell in porcine testes, especially in spermatogonia, spermatocytes, spermatids, Sertoli cells, and Leydig cells (Figure 4A). Subsequently, we labeled the Sertoli cells with SOX9 (the Sertoli cell marker) and confirmed the expression of ALKBH5 in Sertoli cells by IF staining (Figure 4B).

To investigate the functional and regulatory roles of *ALKBH5* in testicular development, we down-regulated *ALKBH5* expression by RNA interference in iSCs. After confirming the silencing effect of *ALKBH5* at the protein expression levels (Figure 4C), we examined the effects of *ALKBH5* silencing on cell viability in iSCs by performing the CCK-8 assay. As shown in Figure 4D, *ALKBH5* silencing induced a strong and significant increase in cell proliferation rates in iSCs (*p* < 0.05). Furthermore, we applied the Annexin-V/PI double staining assays to evaluate the cell apoptosis rates and found that *ALKBH5* silencing significantly inhibits the apoptosis of iSCs (Figure 4E). As BAX promotes apoptosis, and Caspase-3 is the most critical apoptosis executioner protease, we examined the expression levels of these proteins and found that *ALKBH5* silencing inhibits the expression of BAX and Caspase-3 in iSCs as well (Figure 4F). These results suggested that *ALKBH5* plays a central role in iSC cell proliferation and survival.

### 2.7. ALKBH5-Dependent RNA Methylation Modification Regulated SOX9 mRNA m^6^A Level and Gene Expression

**Figure 5:**
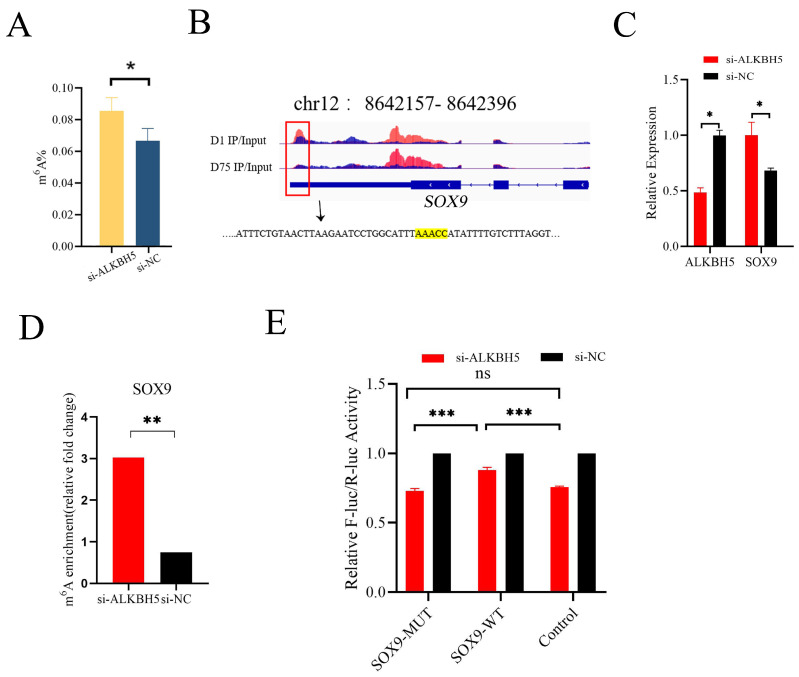
*ALKBH5* regulated the level of RNA methylation and gene expression of *SOX9* mRNA in vitro. (**A**) Total m^6^A levels were measured in iSC-extracted mRNAs after *ALKBH5* silencing. (**B**) Motif analysis using the HOMER program identified “AAACC” as the m^6^A consensus motif in *SOX9* 3′UTR and highlighted by yellow. The red box indicated the m^6^A peak. (**C**) *ALKBH5* silencing significantly up-regulated the mRNA level of *SOX9*. (**D**) MeRIP-qPCR analysis confirmed that *ALKBH5* silencing significantly increased the mRNA m^6^A level of *SOX9*. (**E**) Dual-luciferase assays indicated that the relative fluorescence activities of the *SOX9*-MUT group (mutated m^6^A motif) were significantly lower than those of the *SOX9*-WT group in *ALKBH5* silencing 293T cells. * *p*  <  0.05, ** *p*  <  0.01, *** *p*  <  0.001.

To validate the effects of *ALKBH5* silencing on m^6^A methylation deposition on mRNAs, the total m^6^A levels were measured in mRNAs extracted from iSCs after *ALKBH5* silencing. *ALKBH5* silencing was an effective way to alter the total m^6^A methylation levels in iSCs, as shown in Figure 5A, where a siRNA-induced reduction in *ALKBH5* expression led to a significant increase in the total m^6^A levels.

*SOX9* is a Sertoli-cell-specific marker gene and plays an important role in Sertoli cell differentiation during testicular development. In our MeRIP-Seq data, a peak calling analysis identified an m^6^A peak (located on pig chromosome 12 from 8,642,157 to 8,642,396 bp) in the 3′UTR of *SOX9* mRNA enriched in neonatal pig testis tissue (D1), which was attenuated in pubertal pig testis tissue (D75) (Figure 5B). After *ALKBH5* silencing in iSCs, we found significant increases in *SOX9* mRNA expression (*p* < 0.05) (Figure 5C). By performing MeRIP-qPCR, we confirmed that *ALKBH5* silencing in iSCs resulted in a significant up-regulation of the m^6^A levels of *SOX9* mRNA (Figure 5D). To further validate whether AAACC is the m^6^A motif in *SOX9* 3′UTR, we conducted the dual-luciferase reporter assay. We found that the relative fluorescence activity of the WT group (m^6^A motif) was significantly higher than that of the MUT group (mutated m^6^A motif decreased luciferase activity), indicating *ALKBH5* mediated *SOX9* expression by acting on *SOX9* 3′UTR (Figure 5E).

## 3. Discussion

RNA m^6^A modification is one of the principal post-transcriptional modifications, which is mediated by its effector proteins, including erasers, writers, and readers [17]. Numerous eukaryotic biological and cellular processes are affected by m^6^A, and the correct deposition of m^6^A modifications is required for normal development [18], whereas its dysregulation is implicated in a variety of pathogenic processes [19]. The testis is an essential male reproductive organ, and the production of sperm requires a highly coordinated organization of both testicular germ cells and somatic cells [20]. Almost all types of testicular cells express RNA m^6^A effector proteins, which facilitate dynamic RNA m^6^A modification. Cumulative studies showed a correlation between m^6^A modifications and gene expression patterns in testicular development, spermatogenesis, and male infertility [6]. The development of the testis is a popular research area in reproductive biology; however, the m^6^A modifications that occur during the development of porcine testicular tissue are understudied. Here, we examined the m^6^A transcriptome-wide profile in the testis tissues of Shaziling pigs at birth, puberty, and maturity and defined the correlation between m^6^A modifications and testis-development-related gene expression patterns. Further, we revealed that the m^6^A eraser *ALKBH5* regulated the RNA methylation level and gene expression of *SOX9* mRNA as well as negatively regulated the proliferation of immature porcine Sertoli cells.

Our study found that the global m^6^A level in boar testes from the pubertal (D75) group was distinctly increased compared to that in the neonatal group (D1). The dynamic changes in global m^6^A levels suggested that the up-regulation of m^6^A methylation levels may be required for the initial completion of spermatogenesis in the testes of Shaziling pigs, since immature spermatids were already seen in the testes of pubertal Shaziling boars. Similar results were observed in other independent studies, thus supporting our hypothesis to some extent. For example, the overall m^6^A methylation levels in mature yak testes were significantly higher than those in immature yak testes [12]. In humans, overall m^6^A methylation levels were significantly lower in idiopathic nonobstructive azoospermia patients’ testes, compared with obstructive azoospermia patients’ testes, in which spermatogenesis was unaffected [21].

The methylated m^6^A peaks were highly enriched (~72%) in the 3′UTR and CDS regions at three developmental stages of porcine testes, indicating tissue-specific modifications [22] and agreeing with the peak patterns of cattle testicular development [11], which demonstrated evolutionary conservatism. Generally, m^6^A peaks resided in the 3′UTR and CDS regions, which may affect mRNA stability, translation efficiency, alternative splicing, and maturation [23,24]. Although m^6^A peaks in the testes of neonatal pigs were most enriched in 3′UTR, this enrichment diminished as the boars aged. The genome-wide patterns of m^6^A peaks varied across the developmental stages of pig testes, indicating that the dynamic changes in the regulation of m^6^A methylation were stage-specific.

A total of 7161 unique DMGs were identified in the comparison groups of adjacent testicular development stages. From the functional annotation of DMGs, we found that several key m^6^A-methylated genes were highly enriched in spermatogenesis. For example, *AURKC* has critical roles in the spindle midzone assembly [25]. *OVOL* regulates the meiotic pachytene progression of male germ cells [26]. *SOX8* maintains the microenvironment of the seminiferous epithelium [27]. *ACVR2A* regulates signal transduction and FSH production in the male gland [28]. *SPATA46* is involved in sperm head shaping [29]. We also identified several spermatogenesis-related KEGG pathways. Wnt signaling, which functions in Sertoli cells and postmeiotic germ cells, is needed for several stages of spermatogenesis [30,31]. The cAMP pathway has a unique mechanism that regulates Sertoli cell proliferation in response to the action of FSH [32]. The mTOR pathway is essential for the maintenance of SSC [33] and spermatogonial differentiation [34]. Sertoli cells supply germ cells with energy, while the AMPK signaling pathway regulates intracellular energy [32]. The PI3K/AKT signaling pathway is essential in animals, primarily regulating cell survival, and, therefore, has a major role in the maintenance of spermatogonial stem cell homeostasis [35] and the regulation of Sertoli cell proliferation and apoptosis [32]. The spliceosome-mediated processing of the conversion of pre-mRNA to mRNA is a vital regulatory mechanism in eukaryotes. The testis has the most complex transcriptome and the greatest diversity of alternative splicing among adult animal tissues [36,37]. Thus, precise splicing of mRNA is essential for spermatogenesis [38]. These results suggested that m^6^A methylations were involved in the complex yet well-organized regulation of porcine testicular development and spermatogenesis.

We also identified 4228 unique DEGs among the pairwise groups and found they were significantly enriched in spermatogenesis, spermatid development, flagellated sperm motility, and intracellular signal transduction. Furthermore, the conjoint MeRIP-Seq and RNA-Seq analyses revealed coordinated patterns between m^6^A modifications and gene expression in porcine testes. We focused on resolving the mechanism by which the m^6^A eraser *ALKBH5* regulates *SOX9* (a hypo-down gene in our conjoint analysis) gene expression during porcine testis development.

Studies showed that *ALKBH5* is highly expressed in the testes of mice [9] and goats [39]. As a mammalian RNA demethylase, ALKBH5 is essential for mouse fertility [9]. In our study, we confirmed that ALKBH5 is expressed in porcine testicular Sertoli cells. Sertoli cells are an essential type of somatic cell in the testis because they are in direct contact with germ cells and play a crucial role in regulating spermatogenesis, including maintaining the structure of seminiferous tubules, establishing the blood–testis barrier, and nourishing germ cells in a specific niche environment [40]. The final number of mature Sertoli cells attained during the proliferative phase of immature Sertoli cells determines sperm production capacity because each Sertoli cell can only support a certain number of germ cells [32]. The *SOX9* gene, encoding SRY-box transcription factor 9, is a master regulator and is essential for Sertoli cell differentiation during testicular development [41]. In testes, SOX9 expression is restricted to Sertoli cells and is consistently expressed through adulthood [20]. Therefore, it is crucial to determine whether m^6^A modification can regulate the proliferation of immature Sertoli cells. In this study, we demonstrated that *ALKBH5* negatively regulates the proliferation of immature porcine Sertoli cells. Further, we discovered that *ALKBH5* silencing resulted in a significant increase in *SOX9* mRNA expression. The MeRIP-Seq data further revealed the enrichment of m^6^A peaks in the 3′UTR region of *SOX9* mRNA, with a marked decrease presented in the testicular tissue of pubertal pigs compared to newborn boars. Additionally, MeRIP-qPCR confirmed that *ALKBH5* silencing led to an up-regulation of m^6^A modification levels in *SOX9* mRNA. The dual-luciferase reporter assays further validated AAACC as the m^6^A motif in the 3′UTR of *SOX9* mRNA. Therefore, based on these findings, we inferred that the expression of *SOX9* mRNA was regulated by *ALKBH5*-dependent RNA methylation modification. Through the regulation of m^6^A modification levels, *ALKBH5* may influence the stability, translation efficiency, and post-transcriptional regulation of *SOX9* mRNA. This regulatory mechanism may involve the impact of m^6^A modification on RNA structure and stability as well as the changes in the binding affinity of m^6^A-related proteins. These speculations will be further tested in our subsequent study, which should provide us with more insights into the role of *ALKBH5* in the regulation of *SOX9* gene expression.

In mammalian spermatogenesis, m^6^A modification controls many genes in germ cells and somatic cells in the testes [42,43]. These genes are involved in spermatogonia differentiation, meiosis, spermiogenesis, proliferation, apoptosis, and other processes [10]. The m^6^A modifications in the testis are dynamic and rapidly respond to environmental exposures and pathological factors, such as environmental toxicants [44,45,46], heavy metals [47,48], high-fat diets [49], physical exercises [50], idiopathic nonobstructive azoospermia [21], and male genital system tumors [51]. Pigs are anatomically and physiologically comparable to humans and are well-suited as non-rodent biological models for studying male infertility and testicular tumors due to the rapid rate of sexual maturation in boars. Understanding the changes in m^6^A modifications induced by adverse exposures may serve as molecular biomarkers for monitoring, provide an early diagnosis of negative health outcomes, mitigate undesirable damages, and develop more effective treatment strategies for diseases [51]. In contrast, in response to global environmental changes and pollution, in-depth studies of m^6^A modifications in porcine testes could serve as targets to reduce reproductive disorders in boars, thus contributing to pig production and local breed conservation.

## 4. Materials and Methods

### 4.1. Sample Collection

Testicular tissues were collected from nine half-sib healthy boars (breed: Shaziling) in Xiangtan, Hunan Province, China. Of these, three were 1 day old, three were 75 days old, and three were 150 days old.

### 4.2. RNA m^6^A Dot Blot Assay of Porcine Testicular Tissue

Total RNA (200 ng) was isolated and loaded onto a positively charged nylon transfer membrane. After crosslinking by UV, the membrane was then blocked by 5% BSA for 1 h, followed by incubation with m^6^A monoclonal antibody (Catalog No. 68055-1-Ig, 1:2000; Proteintech, Wuhan, China) at 4 °C overnight. Then, the membrane was incubated with HRP-conjugated goat anti-rabbit IgG at room temperature for 2 h, followed by detection with an ECL imaging system (Millipore, Burlington, Worcester, MA, USA). As a loading negative control, 0.02% methylene blue was utilized.

### 4.3. Target Gene Expression Assay Using RT-qPCR

Total RNAs were extracted from testicular tissues using TRIzol (Invitrogen, Carlsbad, CA, USA) and subjected to the synthesis of cDNA by reverse transcription with the PrimeScript RT Reagent Kit with gDNA Eraser (TAKARA, Beijing, China). The expression levels of RNA methylation-related genes such as *METTL3*, *METTL14*, *WTAP*, *ALKBH5*, *FTO*, *YTHDC1*, *YTHDC2*, *YTHDF1*, *YTHDF2*, and *YTHDF3* were detected using RT-qPCR. Five differentially m^6^A methylated (DMGs) genes (*BAG6*, *SOX9*, *KDM3A*, *PRM2*, and *RARA*) were also randomly selected and analyzed by RT-qPCR to verify the RNA-Seq results. Primers were designed using Primer 5.0 software and synthesized by Sangon Biotech Co., Ltd., (Shanghai, China). RT-qPCRs were performed using a CFX96 Real-Time PCR Detection System (Bio-Rad, Hercules, Los Angeles, CA, USA). The relative gene expression level was normalized to β-actin and calculated using the comparative cycle threshold (Ct) method (2^−ΔΔCT^). The primers used for RT-qPCR are listed in Supplementary Table S5.

### 4.4. MeRIP-Seq and RNA-Seq

For each testis sample, total RNA was extracted and purified using TRIzol (Invitrogen, Carlsbad, CA, USA) and evaluated for quantity and integrity using NanoDrop ND-1000 (NanoDrop, Wilmington, DE, USA) and Bioanalyzer 2100 (Agilent, Santa Clara, CA, USA) with a RIN value > 7.0. Poly (A) mRNA was isolated from 50 μg total RNA using Dynabeads Oligo (dT) 25 (Thermo Fisher, San Jose, CA, USA) and then fragmented at 86 °C for 7 min using the Magnesium RNA Fragmentation Module (NEB, USA). RNA fragments were separated into two different groups. One group was incubated at 4 °C for 2 h with an m^6^A-specific antibody (Synaptic Systems, Göttingen, Germany) in IP buffer containing 50 mM Tris-HCl, 750 mM NaCl_2_, and 0.5% IGEPAL CA-630 to perform m^6^A RNA immunoprecipitation (IP). The other group was used to create an input sample without IP. Sequencing libraries of IP samples (used for MeRIP-Seq) and input samples (used for RNA-Seq) were sequenced on an Illumina Novaseq 6000 sequencer (Illumina, Inc., San Diego, CA, USA) for paired-end sequencing (mode PE150).

### 4.5. Bioinformatic Analysis of MeRIP-Seq and RNA-Seq Data

Raw sequencing data for MeRIP-Seq (IP) and RNA-Seq (Input) were quality controlled using fastp [52], which included the removal of adaptors, duplicate sequences, and low-quality bases to obtain clean data. Using HISAT2 [53], the cleaned reads were mapped onto the pig reference genome (Sscrofa11.1). The R package exomePeak2 [54] was used to call m6A peaks from mapped reads in IP libraries. Using ChIPseeker v1.0 [55] software, the called peaks were annotated by intersection with gene structures. MEME [56] and HOMER [57] were used to perform de novo and known motif searches and locate the motifs with the peaks. Using the exomePeak2 package in R [54], the differential m^6^A peaks with |log_2_ (fold change)| ≥ 1 and *p*-value < 0.05 between the compared groups were evaluated. StringTie [58] was then utilized to calculate the expression level of all mRNAs from input libraries by computing FPKM (total exon fragments/mapped reads (millions) exon length (kb)). By using the R package edgeR [59], mRNAs with |log_2_(fold change)| ≥ 1 and *p*-value < 0.05 were identified as differentially expressed genes (DEGs). Using clusterProfiler [60], Gene Ontology (GO) enrichment and Kyoto Encyclopedia of Genes and Genomes (KEGG) pathway enrichment analyses were carried out on the differential peaks and discovered DEGs, and the plot results were visualized using the ggplot2 package [61] in R. Furthermore, a conjoint analysis of differentially methylated peaks (DMPs) (|log_2_(fold change)| ≥ 1, *p* < 0.05) and gene expression levels (DGEs) (|log_2_(fold change)| ≥ 1, *p* < 0.05) in comparisons between adjacent developmental stages was performed.

### 4.6. Immunohistochemical Staining (IHC) and Immunofluorescence Staining (IF)

Freshly collected testicular samples were fixed in 4% polyoxymethylene, embedded in paraffin, and subsequently cut into 5 mm thick sections. Briefly, the slides were deparaffinized, the antigen was repaired, endogenous peroxidase was inactivated by H_2_O_2,_ and the slides were blocked with serum. For IHC, slides were pretreated and incubated with rabbit anti-ALKBH5 antibody (ab195377; Abcam, Cambridge, UK, 1:500). For IF, pretreated slides were incubated at 4 °C overnight with rabbit anti-ALKBH5 (ab195377; Abcam, Cambridge, UK, 1:200) and mouse anti-SOX9 (1:200) antibodies (67439-1-Ig; Proteintech, Wuhan, China, 1:200). After the slides were incubated with secondary antibodies, DAPI solutions were added to stain the nuclei. The slides were then examined using a laser scanning confocal microscope (Nikon, Tokyo, Japan) at wavelengths of 555 nm (red, ALKBH5), 490 nm (green, SOX9), and 405 nm (blue, DAPI).

### 4.7. ALKBH5 Silencing of Immature Porcine Sertoli Cells

The swine testicular cell line was bought from the American Type Culture Collection (ATCC-CRL-1746), which was originally isolated from pig testes (80–90 days) and characterized as immature porcine Sertoli cells (iSCs) [62]. The iSCs were cultured in Dulbecco’s Modified Eagle’s Medium (DMEM) (Gibco, Detroit, MI, USA) containing 10% fetal bovine serum (FBS) (Gibco, Detroit, MI, USA) and 100 IU/mL penicillin-streptomycin and incubated in a humidified atmosphere at 37 °C with 5% CO_2_.

Three si-*ALKBH5* sequences were designed and synthesized by RiboBio Co., Ltd., (Guangzhou, China), and one of the sequences with the highest silencing efficiency was selected: 5′-AGGACGAGTGCGCCAAGAT-3′. After cell passaging, when iSC cells reached 80% confluence, Lipofectamine 2000 (Invitrogen, USA) was used for si-*ALKBH5* and also for its negative control (si-NC transfection), according to the manufacturer’s instructions. Cells were harvested 24 h after transfection, and gene-silencing efficiency was evaluated by gene expression (the same method described in Section 4.3) and protein level analysis (the same method described in Section 4.10).

### 4.8. Cell Viability

Cell Counting Kit-8 (CCK-8, UEL-C6005; UElandy, Suzhou, China) was used to assess the cell viability of iSCs. After 24 h of transfection, iSCs were seeded into a 96-well plate at a density of 10^4^ cells per well. CCK-8 medium was added to each well and incubated for 4 h at 37 °C. Values were measured at 450 nm using an ELISA plate reader (Molecular Devices, San Jose, CA, USA).

### 4.9. Cell Apoptosis Assay

The iSCs were cultured in 6-well plates and collected in 1.5 mL centrifuge tubes after 24 h of transfection. Apoptosis assays were then performed on a FACSCanto II flow cytometer (Becton Dickinson, Franklin Lakes, NJ, USA) using the AnnexinV/PI Apoptosis Kit (FXP023; 4A Biotech, Beijing, China).

### 4.10. Western Blotting Analysis

The iSCs were harvested and extracted in strong RIPA lysis buffer (Beyotime, Shanghai, China) with 1% PMSF on ice and then denatured for 10 min at 99 °C supplemented with 5×SDS loading buffer. The total protein was separated by a 10% SDS-PAGE (Epizyme, Shanghai, China) and transferred to a PVDF membrane (Beyotime, Shanghai, China). After blocking the membrane with QuickBlock Blocking Buffer (Beyotime, Shanghai, China) at room temperature for 30 min, the membrane was incubated with antibodies at 4 °C overnight. Western blotting (WB) was performed using anti-ALKBH5 (ab195377; Abcam, Cambridge, UK, 1:500), anti-SOX9 (67439-1-Ig; Proteintech, Wuhan, China, 1:3000), anti-Caspase3 (14220; CST, Danvers, MA, USA, 1:1000), anti-Bax (50599-2-Ig; Proteintech Group, Wuhan, China, 1:2000), and anti-β-actin (60004-1-Ig; Proteintech, Wuhan, China, 1:10,000) antibodies. Finally, the secondary antibodies (anti-rabbit A0208; anti-mouse A0216; Beyotime, Shanghai, China, 1:1000) were incubated for another 1 h, and then chemiluminescent detection was performed.

### 4.11. Total m^6^A Abundance Assay

Total RNAs from si-*ALKBH5* iSCs and its negative control (si-NC transfection iSCs) were isolated and evaluated for quality as previously stated. The m^6^A modification amount of total RNA was quantified using the EpiQuik m^6^A RNA Methylation Quantification Kit (P-9005; Epigentek Group Inc., Farmingdale, NY, USA), following the manufacturer’s instructions. In brief, 200 ng of total RNA was put into each assay well, and then different amounts of m^6^A standard control solutions were added. Then, the capture and detection antibody solutions were added to each well. The levels of m^6^A were colorimetrically quantified by reading the absorbance in a microplate spectrophotometer (Multiskan FC, Thermo Scientific, Lenexa, KS, USA) at a wavelength of 450 nm. The amount of m^6^A was calculated based on the standard curve.

### 4.12. Methylated RNA Immunoprecipitation (MeRIP)-qPCR

The EpiQuik CUT&RUN m^6^A RNA Enrichment (MeRIP) Kit (P9018-24; Epigentek, Farmingdale, NY, USA) was used to detect m^6^A modification of a specific gene (*SOX9*) in iSCs. Total RNA was extracted from si-*ALKBH5* iSCs and its negative control, purified, and eluted, followed by cDNA synthesis using the PrimeScript RT master mix kit (RR036A; Takara, Japan). MeRIP-qPCR for *SOX9* was conducted using the following primers: forward primer: 5′-CATCTCTCCCAACGCCATCT-3′ and reverse primer: 5′-TCTCGCTTCAGGTCAGCCTT-3′. The relevant enrichment level of m^6^A was calculated using 2 (^−ΔΔCT^ [normalized RIP/negative control]).

### 4.13. Dual-Luciferase Activity Assay

The dual-luciferase assay was performed using the Dual-Luciferase Reporter Assay System (E1910; Promega, Madison, WI, USA), according to the manufacturer’s instructions. Combining the position of the m^6^A peak in the MeRIP-Seq data and the conserved RRACH sequence of the m^6^A motif in mammals, we predicted that AAACC was the motif sequence of the m^6^A modification on *SOX9* 3′UTR. To validate whether *ALKBH5* could regulate the expression of *SOX9* by recognizing the AAACC m^6^A motif, we amplified the 500 bp sequence of *SOX9* 3′UTR centered on the motif (WT: AAACC), constructed its mutant (MUT: AACCC), and then inserted it into the pmirGLO vector. Briefly, wild-type (WT) and si-*ALKBH5* 293T cells were transfected with pmiRGLO, pmiRGLO-WT-3′UTR, or pmiRGLO-MUT-3′UTR in a 12-well plate. After 48 h of incubation, cells were analyzed with the chemiluminescence apparatus (ThermoFisher Scientific Inc., Denver, CO, USA). Renilla luciferase (R-luc) was used to normalize firefly luciferase (F-luc) activity to evaluate reporter translation efficiency.

### 4.14. Statistical Analysis

Statistical analyses were performed with GraphPad Prism 9.0 (GraphPad Software Inc., San Diego, CA, USA). The data were reported as the mean  ±  standard deviation (SD). Two-tailed Student’s t-test and one-way analysis of variance (ANOVA), followed by Tukey’s multiple comparisons tests, were used to compare significant differences between and among specified groups (*p* value < 0.05 was considered significant).

## 5. Conclusions

We made a comprehensive analysis of the transcriptome-wide m^6^A methylome of porcine testicular development. We further discovered that the m^6^A eraser *ALKBH5* negatively regulated the proliferation of immature porcine Sertoli cells. *ALKBH5* also regulated the level of RNA methylation and gene expression of *SOX9* mRNA in vitro. In addition to serving as potential targets for enhancing boar reproduction, our findings contributed to a greater understanding of the regulation of m^6^A modifications in male reproduction.

## Data Availability

The sequencing data were uploaded to the National Genomics Data Center under GSA accession number CRA010693 (https://ngdc.cncb.ac.cn/gsa accessed on 20 August 2023).

## Supplementary Materials

The supporting information can be downloaded at: https://www.mdpi.com/article/10.3390/ijms241914475/s1.
