# Supplementary material for: Comprehensive Analysis of the Transcriptome-Wide m6A Methylome in Shaziling Pig Testicular Development
Source: Int J Mol Sci. 2023 Sep 23;24(19):14475. doi: 10.3390/ijms241914475 (PMC10572705; doi:10.3390/ijms241914475)
Supplement: Supplementary file 1 [file ijms-24-14475-s001.zip › ijms-2600650-supplementary.pdf]

Supplementary Information

Comprehensive Analysis of the Transcriptome-Wide m^{6}A Methylome in Shaziling Pig Testicular Development

Chujie Chen [1,2], Xiangwei Tang [1,2], Saina Yan [1,2], Anqi Yang [1,2], Jiaojiao Xiang [1,2], Yanhong Deng [1,2], Yulong Yin [1,3], Bin Chen [1,2,*] and Jingjing Gu [1,2,*]

[1] College of Animal Science and Technology, Hunan Agricultural University, Changsha 410128, China;

[2] Hunan Provincial Key Laboratory for Genetic Improvement of Domestic Animal, Hunan Agricultural University, Changsha 410128, China

[3] Key Laboratory of Agro-Ecological Processes in Subtropical Region, Institute of Subtropical Agriculture, Chinese Academy of Sciences, Changsha 410125, China

* Correspondence: chenbin7586@hunau.edu.cn (B.C.); jingjing.gu@hunau.edu.cn (J.G.)

Supplementary Figures

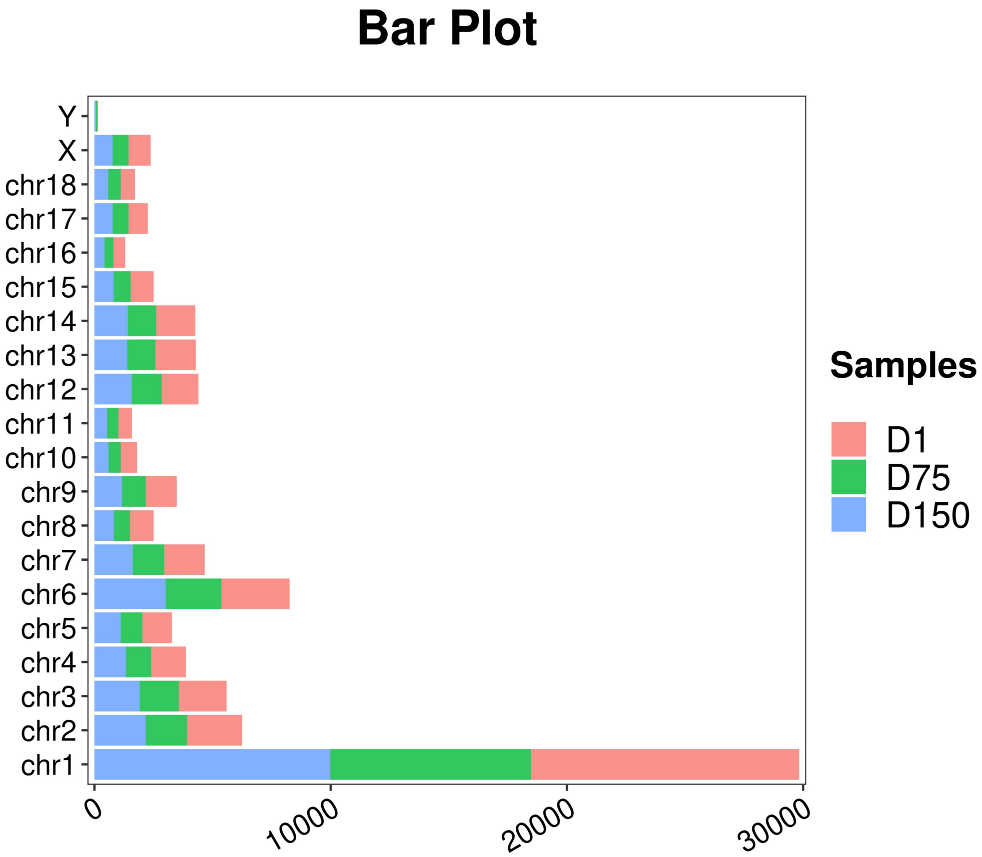

Supplementary Figure S1. Distribution of m^{6}A peaks across pig chromosomes in the three groups (D1, D75 and D150).

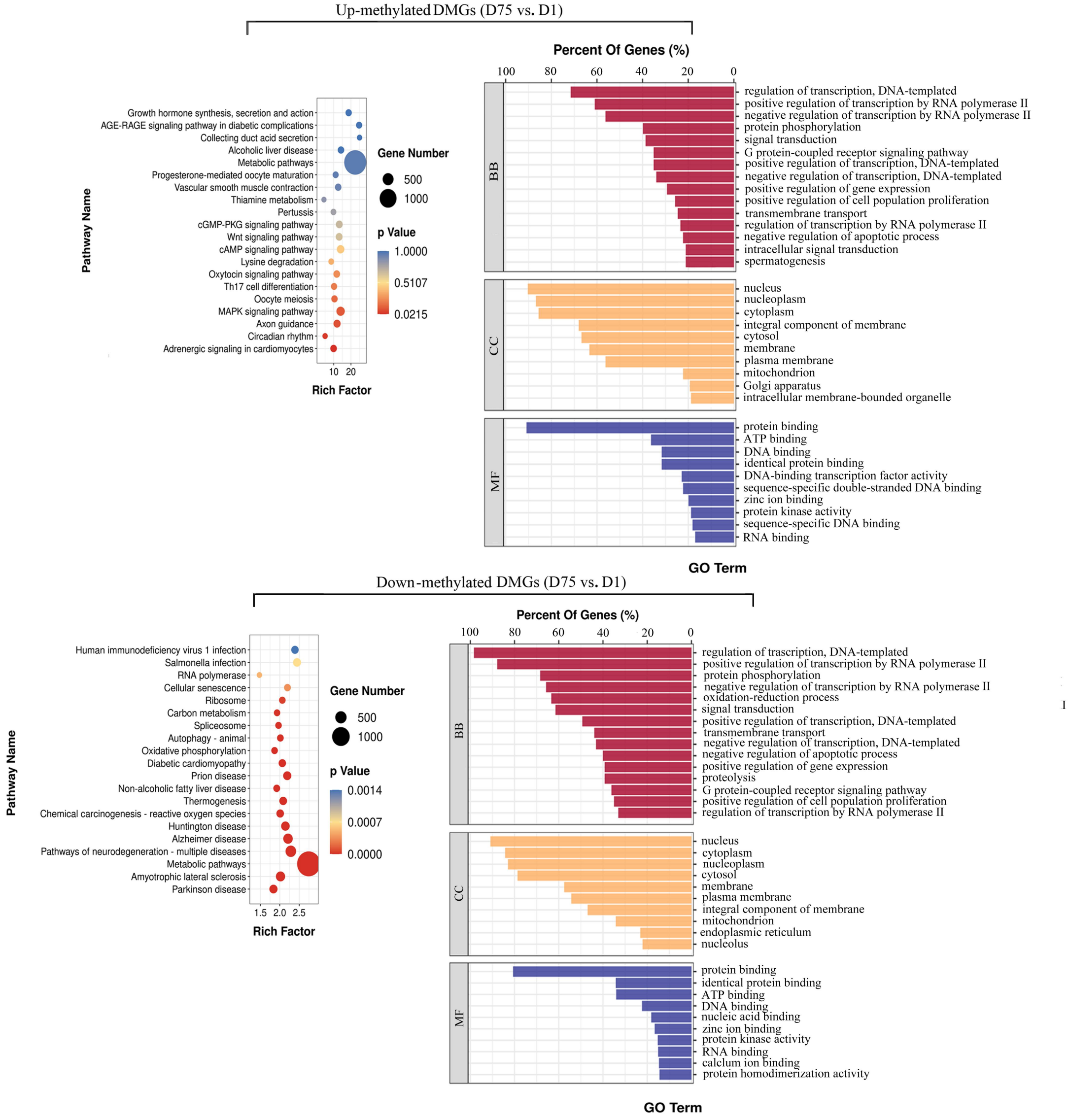

Supplementary Figure S2. GO and KEGG enrichment analysis of up-methylated and down-methylated DMGs for the D75 vs. D1 group.

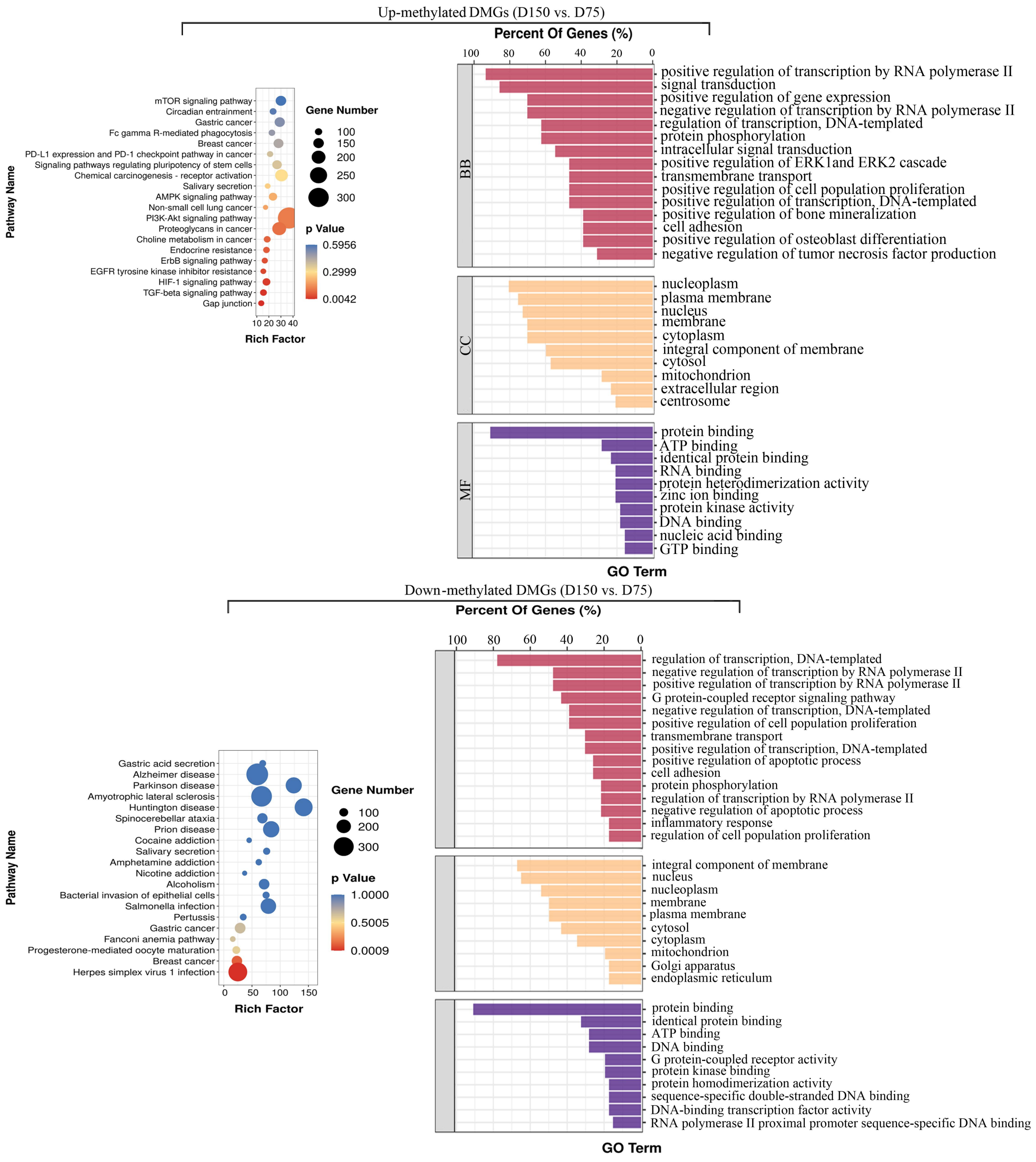

Supplementary Figure S3. GO and KEGG enrichment analysis of up-methylated and down-methylated DMGs for the D150 vs. D75 group.

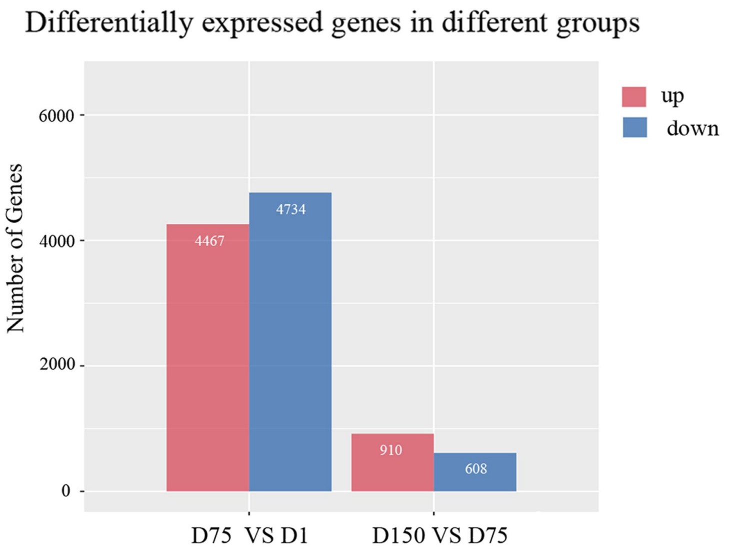

Supplementary Figure S4. Barplots showing the number of up-and down-regulated DEGs between the studied groups.

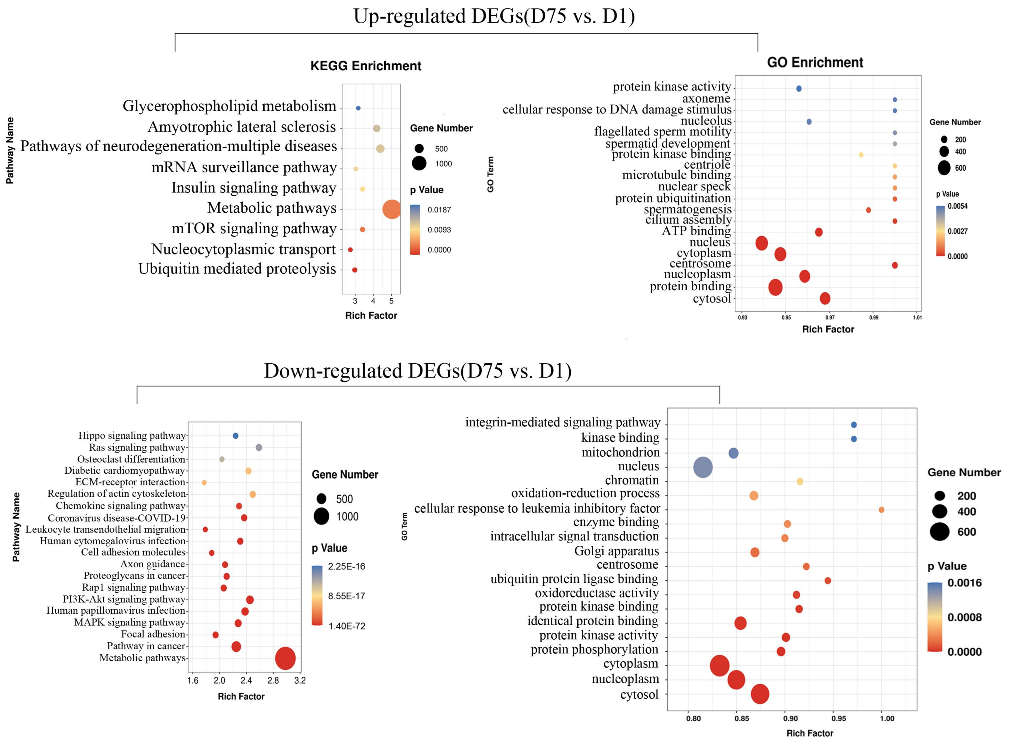

Supplementary Figure S5. GO and KEGG enrichment analysis of up-regulated and down-regulated DEGs for the D75 vs. D1 group.

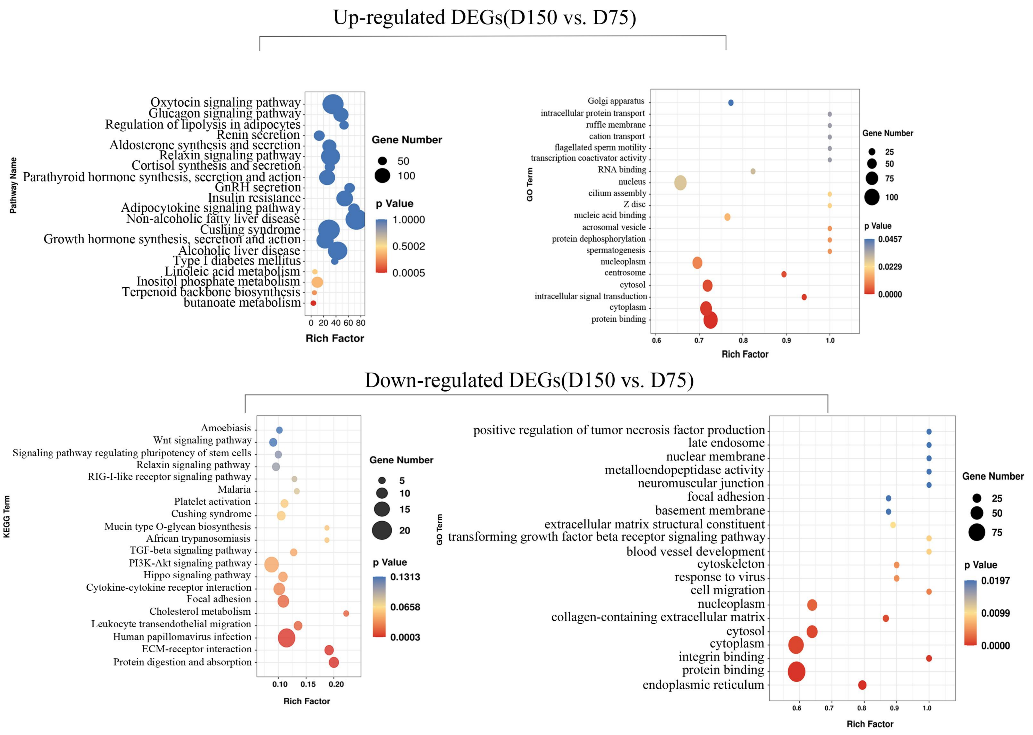

Supplementary Figure S6. GO and KEGG enrichment analysis of up-regulated and down-regulated DEGs for the D150 vs. D75 group.

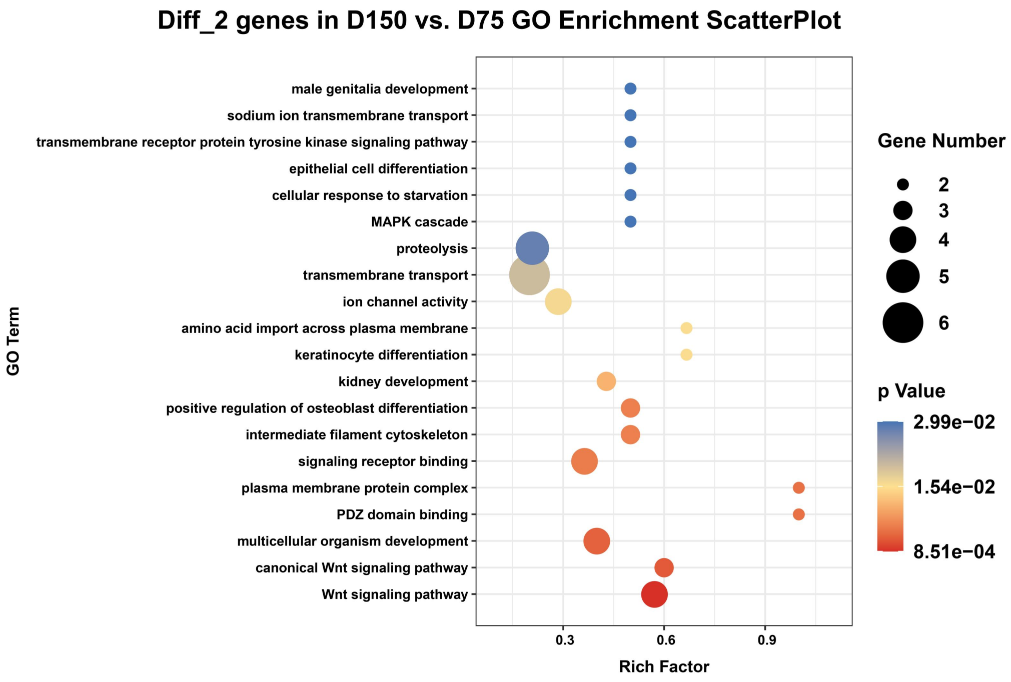

Supplementary Figure S7. GO enrichment analysis of Diff_2 gene sets in D150 vs. D75.

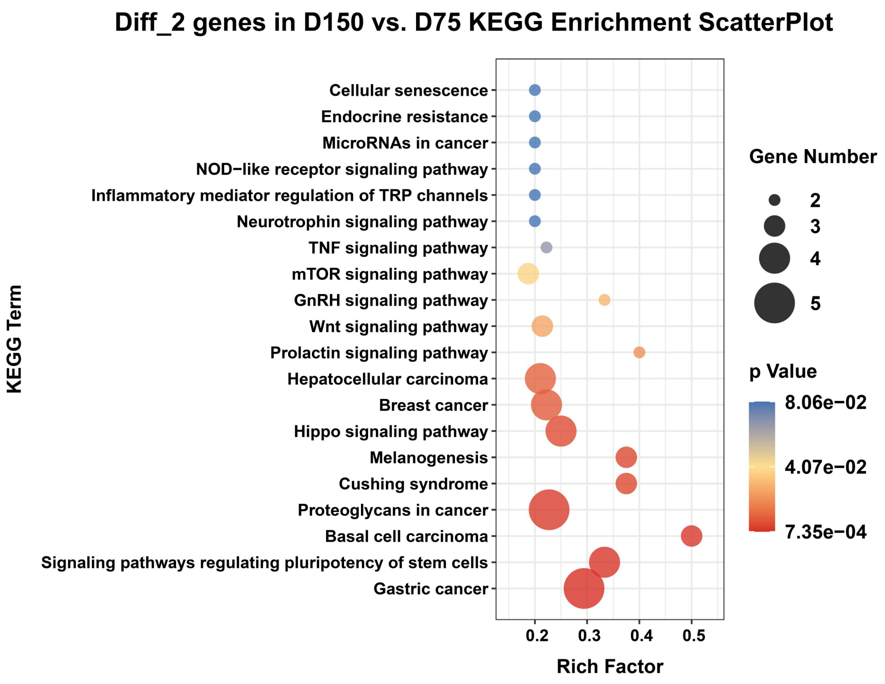

Supplementary Figure S8. KEGG enrichment analysis of Diff_2 gene sets in D150 vs. D75.

Supplementary Tables

Supplementary S1. Statistics of sequence data for MeRIP-Seq and RNA-Seq before and after quality control

Sequence Types	Sample ID	Raw reads (Mb)	Raw bases (Gb)	Clean reads (Mb)	Clean bases (Gb)	Mapping ratio (%)	Q20 (%)	Q30 (%)	GC (%)
MeRIP-Seq (IP)	D1_1_IP	52.72	7.91	48.70	6.62	83.76	97.84	94.04	53.31
	D1_2_IP	54.60	8.19	52.53	7.20	87.88	97.78	93.99	52.61
	D1_3_IP	53.90	8.09	50.30	6.87	84.97	97.73	93.83	54.34
	D75_1_IP	54.37	8.16	49.29	6.73	82.55	97.94	94.27	55.28
	D75_2_IP	43.74	6.56	40.53	5.49	83.65	97.91	94.16	54.66
	D75_3_IP	45.52	6.83	42.23	5.69	83.35	97.91	94.16	55.12
	D150_1_IP	53.95	8.09	50.87	6.93	85.59	97.85	94.06	54.34
	D150_2_IP	47.23	7.08	44.89	6.10	86.08	97.84	94.04	54.21
	D150_3_IP	52.02	7.80	49.95	6.87	88.04	97.93	94.21	54.36
RNA-Seq (Input)	D1_1_input	52.42	7.86	50.68	6.99	88.94	97.91	94.03	51.88
	D1_2_input	48.73	7.31	46.70	6.40	87.61	97.96	94.22	54.40
	D1_3_input	50.41	7.56	48.65	6.72	88.85	97.79	93.80	52.20
	D75_1_input	45.21	6.78	43.46	5.98	88.24	97.94	94.17	53.54
	D75_2_input	52.09	7.81	50.63	7.04	90.04	97.89	93.90	51.00
	D75_3_input	46.04	6.91	44.55	6.18	89.46	97.92	93.97	51.84
	D150_1_input	47.14	7.07	46.05	6.40	90.53	97.84	93.79	50.84
	D150_2_input	44.05	6.61	43.17	6.01	90.96	97.84	93.81	50.86
	D150_3_input	53.54	8.03	52.12	7.25	90.25	97.98	94.14	51.14

Supplementary S2. Statistics of the detected m^6A methylation peaks

**Group Name**	**D1**	**D75**	**D150**
Number of Peaks	26,314	20,011	23,972
Total Length of Peaks (bp)	54,208,044	69,904,930	76,325,047
Average Length of Peaks (bp)	2060	6986	6368
Median Length of Peaks (bp)	242	360	331
Percentage of Genome (%)	2.17	2.79	3.05

Supplementary S3. List of top 0.5% Diff_1 genes with significant changes in m^6A and mRNA transcript abundance in porcine testes (D75 vs. D1)

**Ensembl Gene ID**	**Gene Symbol**	**Pattern**	**m6A level change**					**mRNA level change**	
			**Chr**	**Peak start**	**Peak end**	**diff.log2.fc**	**diff.p**	**log2(fc)**	**qval**
ENSSSCG00000039057	ALKAL2	Hypo-Down	chr3	132669551	132669641	-4.83	0.00	-3.39	0.00
ENSSSCG00000034174	AURKC	Hyper-Up	chr6	61643391	61643872	5.61	0.00	2.46	0.00
ENSSSCG00000036255	CACNG3	Hypo-Up	chr3	21995230	21995410	-5.67	0.00	3.70	0.00
ENSSSCG00000028139	CCDC154	Hyper-Up	chr3	40415031	40415879	4.93	0.00	4.04	0.00
ENSSSCG00000031023	CD24	Hypo-Down	chr1	73184782	73184933	-2.17	0.00	-5.78	0.00
ENSSSCG00000009031	EDNRA	Hypo-Down	chr8	81271049	81271200	-5.04	0.03	-2.32	0.00
ENSSSCG00000015596	FAM71A	Hypo-Up	chr9	130772250	130772550	-1.93	0.00	9.81	0.00
ENSSSCG00000013044	FLRT1	Hypo-Down	chr2	7990865	8052262	-4.94	0.00	-4.02	0.00
ENSSSCG00000038221	HSD17B2	Hypo-Down	chr6	6436658	6437258	-1.87	0.00	-6.28	0.00
ENSSSCG00000000475	IRAK3	Hyper-Down	chr5	30526037	30526278	5.24	0.00	-2.20	0.00
ENSSSCG00000002014	JPH4	Hyper-Up	chr7	75522659	75522809	4.81	0.00	3.90	0.00
ENSSSCG00000001597	LRFN2	Hyper-Up	chr7	35914826	35914976	5.98	0.01	4.01	0.00
ENSSSCG00000008832	LRRC66	Hypo-Down	chr8	39107898	39108108	-5	0.05	-2.55	0.00
ENSSSCG00000035269	NXNL2	Hypo-Down	chr14	356961	357230	-3.47	0.01	-6.41	0.00
ENSSSCG00000017501	PNMT	Hypo-Up	chr12	22649891	22650131	-2.48	0.01	11.28	0.00
ENSSSCG00000032950	SLC25A34	Hypo-Down	chr6	74907588	74907768	-1.84	0.00	-5.90	0.00
ENSSSCG00000031449	SLC27A5	Hyper-Up	chr6	63075441	63076370	2.63	0.00	13.08	0.00
ENSSSCG00000025535	TFAP2B	Hyper-Down	chr7	44755579	44764791	4.78	0.00	-2.64	0.00
ENSSSCG00000011668	TRIM42	Hyper-Up	chr13	81449569	81449839	2.59	0.00	10.45	0.00
ENSSSCG00000040678	TYMS	Hyper-Down	chr6	105660184	105660601	4.82	0.00	-2.13	0.00

Supplementary Table S4. List of top 0.5% Diff_2 genes with significant changes in m^6A and mRNA transcript abundance in porcine testes (D150 vs. D75)

Gene	Symbol	Pattern	m6A level change					mRNA level change	
			Chr	Peak start	Peak end	diff.log2.fc	diff.p	log2(fc)	qval
ENSSSCG00000001027	BMP6	Hyper-Down	chr7	5208983	5209761	1.06	0.00	-2.14	0.00
ENSSSCG00000012959	CATSPER1	Hyper-Up	chr2	6314831	6318023	1.10	0.00	1.40	0.01
ENSSSCG00000016164	IKZF2	Hypo-Up	chr15	115677266	115677417	-4.70	0.00	2.36	0.00
ENSSSCG00000002915	KIRREL2	Hypo-Up	chr6	45278498	45279933	-1.14	0.00	3.56	0.00
ENSSSCG00000035442	SLC36A1	Hyper-Up	chr16	71544806	71548603	1.34	0.00	1.26	0.01
ENSSSCG00000030044	TMEM184C	Hypo-Up	chr8	81115953	81116014	-1.10	0.00	1.12	0.01
ENSSSCG00000023647	WNT8A	Hyper-Up	chr2	140106571	140108946	1.46	0.00	3.25	0.00

Supplementary Table S5. List of primers used for RT-qPCR validation

Gene Name	Forward primer	Reverse primer
METTL3	CTACTGTACCCACTTCTGG	TGTAGGAGACTTCGCTTT
METTL14	GGCTTCTTATGATACCTCTACTCCA	TTCTTCAAATCTGTCGGCCAAAC
WTAP	CTGGAAGTTTACGCCTGAT	TGGTACTCTGCATACCCTCT
ALKBH5	GTGCTTTCCCTGCCTGTGCG	GGTTCTCCTCCTTGTCCATCTCC
FTO	GATCTCAATGCCACCCACCA	CCACTCAAACTCGACCTCGT
YTHDC1	ACCCTTACTATCAGCACC	GTCTACTTCTCCGACCAC
YTHDC2	AGTGGGCGACTCAACAAT	CTTTCAGCCACAGCGATA
YTHDF1	CGTGGACCCCCAGAGAACG	AGTAGCTGGACAAGTAGGGGT
YTHDF2	CAGGCAAGGCCCAATAATGC	TCTCCGTTGCTCAGTTGTCC
YTHDF3	GAGCCTGTCCGCCATTGT	TGCCCTTTAGGTCTCTGATCCA
BAG6	GTCCGCAGGGTTGGTGAT	TCCGCTGGCTCTGAATGT
SOX9	CATCTCTCCCAACGCCATCT	TCTCGCTTCAGGTCAGCCTT
KDM3A	AAGCCGTAAAAACGAAACCT	CGCAGACAGACACACAACAA
PRM2	AGTCCGAGTGAAAGTCCGCAG	TGTGGCTCCTGTGTCTGTAGTGG
RARA	AAGATGTACGAGAGTGTGGACG	CTCTGGGTCTCAATGGAGTGGTT
